# Optimizing the effective doses of mitomycin C, 5-fluorouracil, and their combination on cultivated basal cell carcinoma

**DOI:** 10.5935/0004-2749.20210049

**Published:** 2021

**Authors:** Sahar Balagholi, Maryam Aletaha, Mozhgan Rezaei Kanavi, Samira Karami, Rasul Dabbaghi, Parisa Ashtar Nakhaie

**Affiliations:** 1 Blood Transfusion Research Center, High Institute for Research and Education in Transfusion Medicine, Tehran, Iran; 2 Ophthalmic Research Center, Shahid Beheshti University of Medical Sciences, Tehran, Iran; 3 Ocular Tissue Engineering Research Center, Shahid Beheshti University of Medical Sciences, Tehran, Iran

**Keywords:** Basal Cell Carcinoma, Mitomycin C, 5-Fluorouracil, *TP53*, *CDKN1A*, *CDK6*, Carcinoma basocelular, mitomicina C, 5fluorouracil, *TP53*, *CDKN1A*, *CDK6*

## Abstract

**Purpose:**

This study aimed to optimize the effective doses of mitomycin C, 5-fluorouracil, and their combination on cultivated basal cell carcinoma.

**Methods:**

Cultivated basal cell carcinoma and fibroblastic cells were treated with different concentrations of mitomycin C, 5-fluorouracil, and their combination. Cell viability, cell cycle, apoptosis, and expression levels of *TP53, CDKN1A*, and *CDK6* were investigated. The most effective drug with its optimum dosage was administered via multiple intralesional injections to a 65-year-old woman with advanced periorbital nodulo-ulcerative BCC.

**Results:**

The concentrations of 0.00312 and 0.312 mg/mL were considered optimum for mitomycin C and 5-fluorouracil, respectively. The mean viabilities of basal cell carcinoma treated with mitomycin C alone and its combination with 5-fluorouracil were significantly less than those of the controls (p=0.002 and p=0.04, respectively). The cell cycle of all the treated basal cell carcinoma groups was arrested in the S phase. The apoptotic rates (p=0.002) of mitomycin C treated basal cell carcinoma were higher than those of the other treated cells, and their *TP53* was significantly upregulated (p=0.0001). Moreover, *CDKN1A* was upregulated, whereas *CDK6* was downregulated in basal cell carcinoma treated with either 5-fluorouracil (p=0.0001 and *p*=0.01, respectively) or the combination of 5-fluorouracil and mitomycin C (p=0.007 and p=0.001, respectively). Basal cell carcinoma lesions were significantly alleviated following mitomycin C injections in the reported patient.

**Conclusion:**

Our in vitro results revealed that the effective doses of mitomycin C and 5-fluorouracil on cultivated basal cell carcinoma were optimized. Mitomycin C was more effective in inducing the apoptosis of basal cell carcinoma than 5-fluorouracil and their combination. The intralesional injections of the optimum dose of mitomycin C could be proposed for the nonsurgical treatment of advanced eyelid basal cell carcinoma.

## Abstract (pt)

**Objetivo:**

Otimizar a dose efetiva de mitomicina C, 5fluorouracil e da combinação de ambos em culturas de células de carcinoma basocelular (CBC).

**Métodos:**

Culturas de células de células de carcinoma basocelular e de fibroblastos foram tratadas com diferentes concentrações de mitomicina C, 5fluorouracil e combinação de ambos. Além disto, foram investigados a viabilidade celular, o ciclo celular, a apoptose e a expressão dos genes *TP53, CDKN1A* e *CDK6*. O medicamento mais eficaz, em sua dosagem otimizada, foi administrado em últiplas injeções intralesionais em uma mulher de 65 anos com carcinoma basocelular nódulo-ulcerativo periorbital avançado.

**Resultados:**

A concentração de 0,00312 mg/mL de mitomicina C e a de 0,312 mg/mL de 5fluorouracil foram consideradas as ideias. A viabilidade média das células de carcinoma basocelular tratadas com mitomicina C isoladamente e em combinação foi significativamente menor que nas células de controle (respectivamente, p=0,002 e *p*=0,04). Todos os grupos de carcinoma basocelular tratados demonstraram interrupção do ciclo celular na fase S. As células de carcinoma basocelular tratadas com mitomicina C mostraram maiores taxas de apoptose (p=0,002) e significativa regulação positiva do gene *TP53* (p=0,0001). Além disso, o gene *CDKN1A* foi po sitivamente regulado e o gene *CDK6* foi negativamente regu lado em células de carcinoma basocelular tratadas com 5fluorouracil (respectivamente, p=0,0001 e p=0,01) ou com a combinação de medicamentos (respectivamente, p=0,007 e p=0,001). Injeções posteriores de mitomicina C na paciente em questão levaram à melhora significativa da lesão do carcinoma basocelular.

**Conclusão:**

Nossos resultados *in vitro* otimizaram as doses efetivas de mitomicina C e 5fluorouracil na cultura de células de carcinoma basocelular e mostraram que a mitomicina C tem mais eficácia na apoptose de células de carcinoma basocelular do que o 5fluorouracil e a combinação de ambos. Injeções intra lesionais de doses otimizadas de mitomicina C podem ser propostas para o tratamento não cirúrgico do células de carcinoma basocelular avançado de pálpebra.

## INTRODUCTION

Basal cell carcinoma (BCC) is known as the most common type of periocular skin cancer; it predominantly affects the lower eyelids of patients aged between 50 and 80 years. Genetic factors, UV radiations, light skin complexion, immunosuppression, aging, and arsenic exposure are the possible risk factors of this malignancy^(1)^. This lesion is mainly treated via surgical excision, which is associated with a low rate of local recurrence; however, the recurrence rate in cases with incomplete excision or medial canthal location may be relatively high within the first 2 years of surgery^(2-5)^. In some instances, such as locally advanced BCC, metastatic tumors, tumor recurrences or post-surgical cosmetic burden, and nonsurgical modalities, including oral administration of sonic hedgehog inhibitors, cryotherapy, photodynamic therapy, radiotherapy, and chemotherapy, may be indicated^(6-11)^. Among chemotherapeutic agents that can reduce the cell cycle and induce apoptosis, 0.02%0.04% mitomycin C (MMC) as an alkylating agent and 5% 5-fluo rouracil (5-FU) as an antimetabolite have been easily used via topical applications or intralesional injections in various superficial skin cancers^(4)^; they also have high rates of tumor eradications^(12-22)^. However, the optimum dosages of these drugs for the treatment of BCC have not been determined. Studies have yet to establish whether a combination of the two drugs will increase their anticancerous properties.

This study was conducted to optimize the effective doses of MMC, 5-FU, and their combination against cultivated BCC cells compared with those against cultured fibroblasts and untreated cells. Additionally, a case with recurrent advanced periorbital BCC treated with the optimum dose of the most effective tested drug was reported.

## METHODS

Full ethical approval was obtained from the ethics committee of the Ophthalmic Research Center, Shahid Beheshti University of Medical Sciences (ORC-SBMU), Tehran, Iran. Signed informed consent was obtained from the patients enrolled in this study in accordance with the principles of the Declaration of Helsinki.

### Culture of BCC Cells

Fresh specimens were obtained from three patients with histopathologically proven nodular BCC, kept at 4°C on a wet gauze, and transferred immediately to a Petri dish containing trypsin at the cell culture laboratory of ORC-SBMU. Four to six rounds of trypsin digestion were carried out at 37°C with 5% CO_2_ for 10 min each. After each round, minced specimens were allowed to settle. Then, supernatants were harvested, supplemented with 20% fetal bovine serum (FBS, GIBCO-BRL, Eggenstein, Germany), and stored at 4°C. At the end of trypsin digestion, pooled supernatants were gently centrifuged at 1,060 × *g* for 5 min. The obtained pellets of the cells were resuspended in a T25 flask containing Dulbecco’s modified Eagle’s medium and Ham’s F12 (DMEM/F12; GIBCO-BRL, Eggenstein, Germany) and 20% FBS and incubated at 37°C in a humidified atmosphere containing 5% CO_2_. All the experiments were performed using the cultivated BCC cells at 80% confluency and at passages 2 and 3. BCC cells were then immunocharacterized for the expression of BER-EP4 and the lack of epithelial membrane antigen (EMA) expression.

### Culture of fibroblasts

Fresh dermis specimens were obtained from blepharoplasty cases and subjected to trypsin digestion as described earlier. Histopathological examination showed that all the dermal specimens had normal dermal structures. The extracted cell suspensions were transferred to T25 flasks containing DMEM/F12 and 20% FBS and incubated at 37°C in a humidified atmosphere containing 5% CO_2_. In all the experiments, cultivated fibroblasts at 80% confluency and at passages 2 and 3 were used. The fibroblasts were then immunocharacterized for vimentin expression.

### Immunocytochemistry

The cultivated BCC and fibroblastic cells were fixed with methanol for 10 min at -10°C and permeabilized with 0.25% Triton X-100 (Sigma-Aldrich, Munich, Germany) and blocked with 1% bovine serum albumin in phosphate buffered saline (PBS) for 90 min at room temperature. The BCC cells were initially incubated with anti-BER-EP4 antibody (1:50, mouse IgG anti-Human antibody; Dako, Carpinteria, CA) and anti-EMA antibody (1:100, mouse monoclonal, Dako, Carpinteria, CA) overnight at 4°C. They were subsequently incubated with fluorescein isothiocyanate (FITC)-conjugated goat anti-mouse IgG (1:100, Santa Cruz Biotechnology) and FITC-conjugated goat anti-mouse IgG (1:100; Santa Cruz Biotechnology) for 45 min in the dark and at room temperature, respectively. The cultivated fibroblasts were incubated with anti-vimentin antibody (1:200, rabbit polyclonal IgG; Santa Cruz Biotechnology Inc., Dallas, USA) overnight at 4°C and treated with the FITC-conjugated goat anti-rabbit IgG (1:100; Santa Cruz Biotechnology Inc., Dallas, USA). After nuclear DNA staining with 4,6-diamidino-2-phenylindole dihydrochloride (DAPI, 1.5 mg/mL; Santa Cruz Biotechnology) was performed, the slides were examined using a fluorescence microscope (Olympus IX71; Tokyo, Japan) at an excitation wavelength ranging from 450 nm to 520 nm. The corresponding images were then captured with a digital camera (Olympus U-TV0.63XC; Tokyo, Japan).

### MTT Assay

The cell viability of the cultivated BCC and fibroblastic cells was assessed using an MTT assay by passing 72h from exposure to different dilutions of MMC and 5-FU. Briefly, the cultured BCC cells and fibroblasts in passages 2 and 3 and with a density of 3 × 10^5^ cells/well were seeded on a 96-well plate and then exposed to different dilutions of MMC (0.025, 0.0125, 0.00625, 0.00312, and 0.00156 mg/mL) and 5-FU (2.5, 1.25, 0.625, 0.312, and 0.156 mg/mL). Then, each well was incubated with 3-(4,5-dimethylthiazol-2-yl)-2,5-diphenyltetrazolium bromide (0.5 g/mL; Sigma-Aldrich, Munich, Germany) for 4h to obtain a purple precipitate. After the culture medium was replaced with 100 mL of dimethyl sulfoxide (Merck, Darmstadt, Germany) and incubated at room temperature for 2h in the dark, the absorption of the samples was read at 540 nm by using an ELISA reader (ELx 808 Absorbance Reader, BioTek Instruments, Winooski, VT). The viability rates of the exposed cells were then compared with the nonexposed controls, and the assay was performed thrice. The optimal dosages of MMC and 5-FU were determined on the basis of the survival of less than 50% of the exposed BCC cells and more than 50% of the exposed fibroblastic cells^(23)^.

### Cell Cycle Investigations

The cellular DNA content was analyzed through flow cytometry and propidium iodide (PI) staining in accordance with previously described methods^(24)^ to investigate the inhibitory effects of MMC, 5-FU, and their combination on the cell cycle steps. Briefly, the cultured BCC cells at a density of 3 × 10^5^ cells per well were plated in 24-well plates and incubated with MMC (0.00312 mg/mL), 5-FU (0.312 mg/mL), and the combination of MMC (0.00312 mg/mL) and 5-FU (0.312 mg/mL) for 72 h. Afterward, the cells were removed via trypsinization and washed with PBS at 3,000 × *g* for 5 min. After the cellular sediments were fixed with ice-cold 80% ethanol (Merck), the cells were kept overnight at -28°C. The cells were recentrifuged at 3,000 × *g* for 10 min at 4°C, washed twice, and incubated with equal amounts of PBS (258 µL) and phosphate citrate buffer (258 µL; Sigma-Aldrich, Saint Louis, MO, USA) for 5 min at room temperature. Then, the cellular sediments were incubated with RNase A (5 µL; Sigma-Aldrich, Saint Louis, MO, USA) and PI (58 µL; Roche, Roche Diagnostics GmbH, Mannheim, Germany) at room temperature in the dark for 30 min. The PI-stained DNA contents of the cells were determined through flow cytometry (BD FACS Calibur, BD Biosciences, San Jose, CA, USA). The final results were analyzed using FlowJo (https://www.flowjo.com/solutions/flowjo/downloads).

### Apoptosis Investigations

The effects of MMC, 5-FU, and their combination on the apoptosis of the cultured BCC cells were investigated through flow cytometry by using annexin V staining. After the cultivated BCC cells at a density of 3 × 10^5^ cells per well were incubated with MMC (0.00312 mg/mL), 5-FU (0.312 mg/mL), and the combination of MMC (0.00312 mg/mL) and 5-FU (0.312 mg/mL) for 72 h, the trypsinized cells were transferred to 15 mL falcon tubes and centrifuged at 3000 × *g* for 5 min at 4°C. The cellular sediments were washed with PBS and incubated with 1X reaction buffer and annexin V (5 µL; IQ Products BV, Rozenburglaan, Netherlands) for 20 min at room temperature in the dark. After 400 µL of 1X reaction buffer was added to each sample, the apoptotic cells were detected through flow cytometry (BD FACS Calibur, BD Biosciences, San Jose, CA, USA) in the cells stained with fluorescein isothiocyanate-labeled annexin V.

### RNA Extraction and Gene Expression Investigations

The cells were cultured in 25 cm^2^ flasks, and total RNA was extracted from the exposed and nonexposed control groups to analyze the gene expression levels of tumor protein 53 (*TP53*), cyclin-dependent kinase inhibitor 1A (*CDKN1A*), and cyclin-dependent kinase 6 (*CDK6*) in cultivated BCC cells exposed to MMC, 5-FU, and their combination. Briefly, the cells were lysed using TRIzol reagent (Life Technologies Corporation, Carlsbad, CA). After chloroform and isopropanol were added, RNA precipitate was obtained and dissolved in nuclease-free water. The purity and concentration of RNA were checked using a NanoDrop spectrophotometer (Thermo Fisher Scientific; Wilmington, DE; A260/280 values and concentrations). Agarose gel electrophoresis was performed on the isolated RNAs to assess the integrity of 28S and 18S rRNA bands. Subsequently, the extracted RNAs were reverse transcribed to cDNA by using a reverse transcriptase kit (Promega, USA) and oligo (dT) primers. The expression levels of *TP53, CDKN1A*, and *CDK6* were determined via quantitative real-time reverse transcriptase polymerase chain reaction (PCR) with EvaGreen master mix (Solis BioDyne, Tartu, Estonia) and specific primers. The PCR parameters were as follows: one amplification cycle for 10 min at 95°C, followed by 40 cycles of denaturation, amplification, and quantification (95°C for 15 s, 58°C-64°C for 30 s, and 72°C for 25 s); the melting curve was initially set at 65°C and gradually increased up to 95°C. The expression levels were normalized by comparing them with the levels of glyceraldehyde-3-phosphate dehydrogenase as an internal control. Relative changes in gene expression were analyzed using 2^-∆∆CT^ method in accordance with the standard curve and efficiency that were established for each primer set. All the assessments were performed thrice, and each sample was run and examined twice.

### Statistical analysis

Nonparametric Kruskal-Wallis and multiple comparison tests were conducted to calculate the differences in cell viability and gene expression levels between study groups. Differences were considered significant when *p*<0.05.

## RESULTS

### Cultivated BCC and Fibroblastic Cells

**Figure 1:**
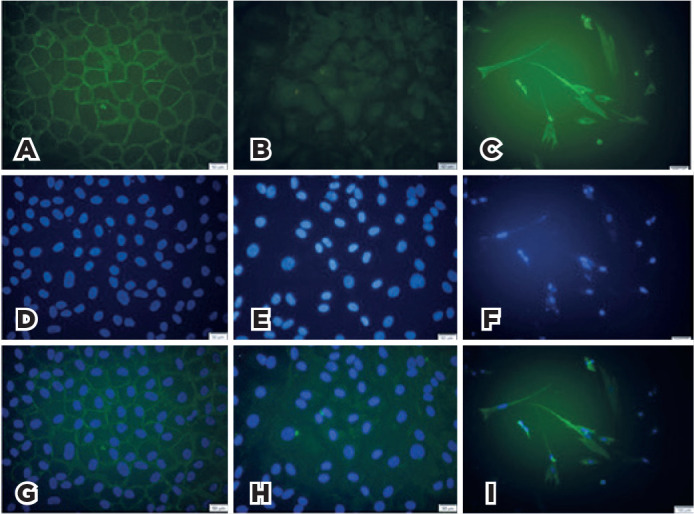
Immunocytochemistry of cultivated BCC and fibroblastic cells. Note the prominent immune reactivity of cultivated BCC cells with BER-EP4, as evidenced by the positive (green) stain of the cell membranes with the FITC-conjugated BER-EP4 antibody (A) and the lack of immune reactivity with the FITC-conjugated EMA antibody (D). The cultivated fibroblastic cells show immune reactivity with the FITC-conjugated vimentin antibody (G). DAPI (blue)-stained cell nuclei (B, E, and H) and merged images (C, F, and i) were illustrated.

Human BCC cells had an epithelioid form, which was apparently different from fibroblastic cells. The BCC cells were immune reactive with BER-EP4 but not with the EMA marker. Fibroblastic cells were immune reactive with vimentin (Figure 1).

### Cell Viability

**Figure 2:**
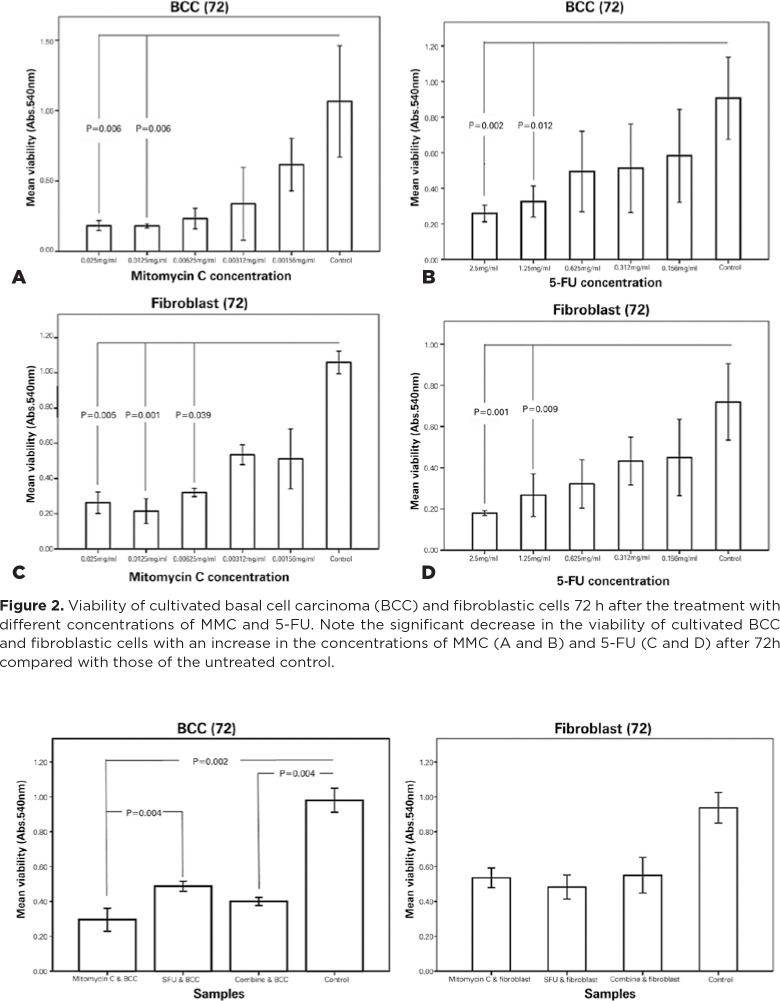
Viability of cultivated basal cell carcinoma (BCC) and fibroblastic cells 72 h after the treatment with different concentrations of MMC and 5-FU. Note the significant decrease in the viability of cultivated BCC and fibroblastic cells with an increase in the concentrations of MMC (A and B) and 5-FU (C and D) after 72h compared with those of the untreated control.

Both cultivated BCC and fibroblastic cells showed a significant reduction of viability and an increase in the concentrations of MMC and 5-FU (Figure 2). The optimal dosages of MMC and 5-FU based on the survival of less than 50% of the exposed BCC cells and more than 50% of the exposed fibroblastic cells, were 0.00312 and 0.312 mg/mL, respectively.

**Figure 3:**
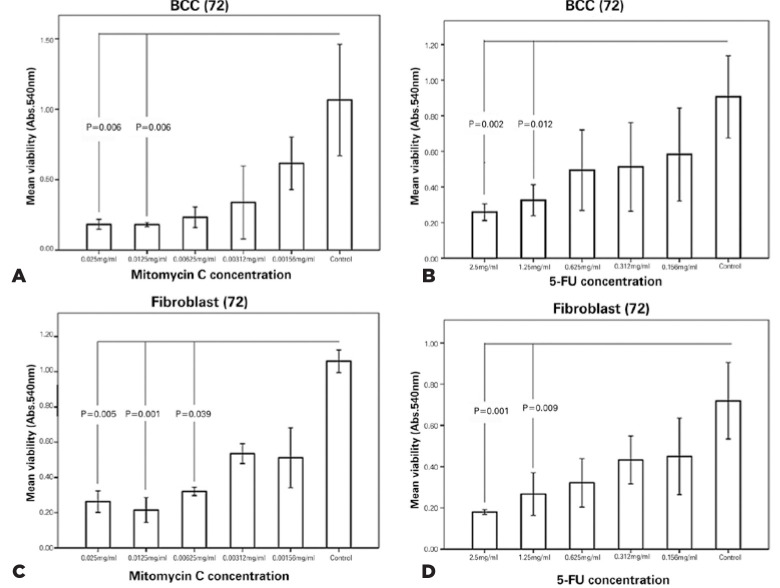
Viability of cultivated basal cell carcinoma (BCC) and fibroblastic cells 72h after the treatment with optimal dosages of MMC, 5-FU, and their combination. Note the significant reduction of the viability of BCC cells 72h after the treatment with optimal dosages of MMC and the combination with MMC and 5FU compared with those of control group (*p*=0.002 and *p*=0.04, respectively). The reductions in the viability of fibroblastic cells 72h after the treatment with the optimal dosages of MMC, 5-FU, and their combination are not significantly different.

In the next step, the viability of the cultivated BCC and fibroblastic cells exposed to the optimal dosages of MMC, 5-FU, and their combination was investigated. The mean viability rates of the exposed fibroblastic cells were not significantly different, but mean viability rate of the BCC cells exposed to MMC alone and the combination of MMC and 5-FU significantly differed from that of the controls (*p*=0.002 and *p*=0.04, respectively; Figure 3). The mean viability of the treated BCC cells with 5-FU alone was not significantly different from that of the controls. The mean viability rate of the BCC cells exposed to the optimal dose of MMC was less than those exposed to the optimal dose of 5-FU (*p*=0.04).

### Cell cycle results

**Figure 4:**
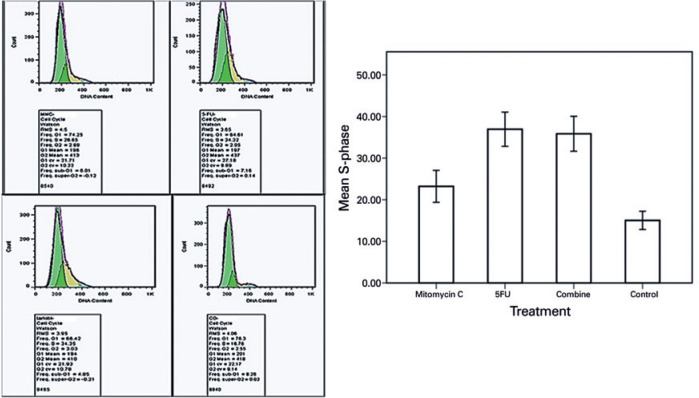
Representative image and corresponding graph of the cell cycle of cultivated BCC cells 72 h after the treatment with optimal dosages of MMC, 5-FU, and their combination. Note the higher rate of S phase cell cycle arrest in the cultivated BCC cells treated with 5-FU alone (36.94% ± 3.57%), MMC alone (23.22% ± 3.33%), and their combination (35.85% ± 3.64%) than that of the untreated controls (15.03% ± 1.90%).

The rate of the S phase cell cycle arrest in the cultivated BCC cells treated with 5-FU (36.94% ± 3.57%), MMC (23.22% ± 3.33%), and their combination (35.85% ± 3.64%) was higher than that of the nontreated controls (15.03% ± 1.90%; Figure 4); however, this difference was not statistically significant (*p*>0.05).

### Cellular apoptosis

**Figure 5:**
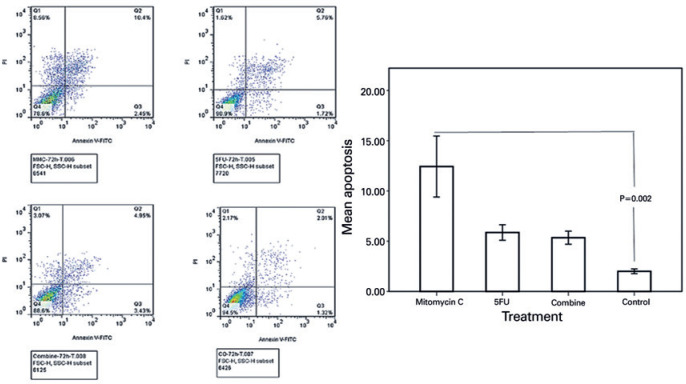
Representative image and corresponding graph of the flow cytometry of cultivated BCC cells for apoptosis 72 h after the treatment with optimal dosages of MMC, 5-FU, and their combination. Note the apoptotic rates of cultivated BCC cells treated with MMC alone (12.43% ± 2.63%), 5-FU alone (5.87% ± 0.67%), and their combination (5.35% ± 0.57%). The apoptotic rate of the treated BCC cells with MMC is significantly higher than that of the untreated controls (2.00% ± 0.20%; *p*=0.002).

The highest rate of apoptosis was observed in the cultivated BCC cells treated with MMC (12.43% ± 2.63%), and this value was significantly different from that of untreated controls (2.00% ± 0.20%; *p*=0.002). The apoptotic rates in the BCC cells treated with 5-FU (5.87% ± 0.67%) and the combination of MMC and 5-FU (5.35% ± 0.57%) were less than that in the BCC cells treated with MMC. Conversely, they were more than the untreated controls (Figure 5), but these differences were not statistically significant (*p*>0.05 in the corresponding comparisons).

### Gene Expression Profile

**Figure 6:**
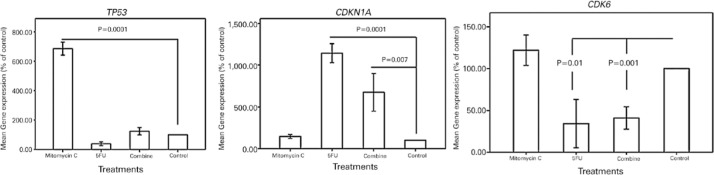
Gene expression analysis of cultivated BCC cells treated with the optimal dosages of MMC, 5-FU, and their combination. Illustrated graphs depict the gene expressions of *TP53, CDKN1A*, and *CDK6* in the cultivated BCC cells treated with the optimal dosages of MMC, 5-FU, and their combination compared with those of the controls (untreated cells). Note the high expression of *TP53* in cultivated BCC cells treated with MMC compared with that in the controls (*p*=0.0001). The expression of *CDKN1A* in cultivated BCC cells treated with 5-FU alone and its combination with MMC is significantly higher than that in the controls (*p*=0.0001 and *p*=0.007, respectively). Note the low expression of *CDK6* in the BCC cells treated with 5-FU alone and its combination with MMC (*p*=0.01 and *p*=0.001, respectively). *TP53*: tumor protein 53; *CDKN1A*: cyclin-dependent kinase inhibitor 1A; *CDK6*: cyclin-dependent kinase 6.

The expression of *CDKN1A* in the cultivated BCC cells treated with 5-FU and the combination of MMC and 5-FU was higher than that in the controls (*p*=0.0001 and *p*=0.007, respectively). The expression of *TP53* in the cultivated BCC cells treated with MMC was also higher than that in the controls (*p*=0.0001). The expression of *CDK6* in the BCC cells treated with 5-FU and the combination of MMC and 5-FU significantly reduced compared with that in the controls (*p*=0.01 and *p*=0.001, respectively; Figure 6).

### Report of a Case

**Figure 7:**
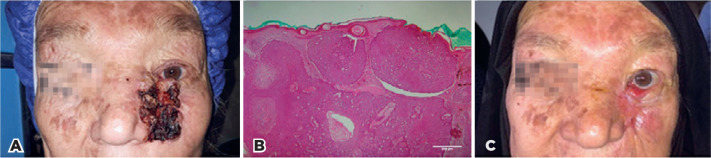
Clinical and histopathological images of the reported patient. Note the presence of an extensive BCC extending from the left medial canthal and the lower lid region to the left nasal ala and the adjacent cheek before intralesional injections of MMC (A). Significant improvement in the lesion, together with a cicatricial left lower lid retraction after the intralesional injections (C). Image B depicts the histopathological features of BCC on the incisional biopsy of the lesion before the intralesional injections of MMC.

A 65-year-old woman with a prior history of left lower lid BCC presented with an advanced ulcerative and verrucous hemorrhagic lesion that had not been definitively treated for over 2 years after the initial biopsy of primary BCC. The patient missed further treatments planned for her residual tumor. The recurrent tumor was an extensive lesion extending from the left medial canthal and the lower lid region to the left nasal ala and the adjacent cheek (Figure 7). In accordance with the 7^th^ edition of the eyelid carcinoma classification system from the American Joint Committee on Cancer^(25)^, the patient’s tumor was diagnosed and regarded as T3aN0M0.

The complete resection of lesions was associated with extensive anatomical damage requiring advanced reparative plastic surgery. As such, a diagnostic incisional biopsy of the lesion concurrent with intralesional injection of MMC (0.00312 mg/mL) was performed after the informed consent was obtained from the patient, considering the patient’s poor socioeconomic conditions. Histopathological examinations revealed the presence of an ulcerative nodular BCC, and the intralesional injections were repeated for the next 2 months (one injection per month). Clinical examinations in the 1^st^ week and by passing 4 months from the 3rd intralesional MMC injection (Figure 7), the lesion significantly improved without evidence of recurrence; however, a cicatricial left lower lid retraction occurred.

## DISCUSSION

To the best of our knowledge, this study is the first to conduct an in vitro examination to determine the optimal doses of MMC, 5-FU, and their combination for cultivated human BCC cells. Our results showed that the rate of apoptosis induced by 0.00312 mg/mL MMC in BCC cells was higher than that caused by 0.312 mg/mL 5-FU and the combination of MMC and 5-FU. These optimal doses were associated with at least 50% viability of the cultivated fibroblastic cells. As such, MMC with a dosage of 0.00312 mg/mL could be used for the local treatment of eyelid BCC even though it could elicit minimal toxic effects on intact surrounding tissues. The clinical outcome after intralesional injections of the optimum dose of MMC in the presented patient also supported our in vitro results.

In addition to the increased expression of *TP53*, apoptosis could be more effectively induced by MMC (0.00312 mg/mL) than 5-FU (0.312 mg/mL) and the combination of MMC with 5-FU, as shown by the results of cell viability and flow cytometry. Moreover, MMC exerted less effect on cell cycle arrest probably because of the preventive effect of p53 on cell cycle complexes^(26,27)^. In the present study, the expression of *CDKNIA*, as an inhibitory gene of cell cycle progression complexes at G1, increased in the BCC cells treated with 5-FU (0.312 mg/mL). Although the results of cell cycle investigations were considered the indicators of a significant cell cycle arrest at S phase in the BCC cells treated with 5-FU, the results of cell viability tests showed a less mortality rate of 5-FU-treated BCC cells than those treated with MMC.

In other studies on human colorectal adenocarcinoma cell lines, a low dose of 5-FU (<100 ng/mL) induces the G2-M phase arrest and mitotic catastrophe, which is a type of cell death resulting from premature or inappropriate entry of cells into mitosis because of physical or chemical stress^(28)^; conversely, a high dose of 5-FU (1000 ng/mL) causes the G1-S phase arrest and apoptosis^(29)^. Given that the optimal dose of 5-FU in our study (0.312 mg/mL) was equivalent to the high dose of 5-FU administered by Yoshikawa et al.^(29)^, the rate of mitotic catastrophe of the treated BCC cells was higher than that of apoptosis. This result also indicated that the rate of apoptosis induced by 5-FU was lower than that of MMC in the cultivated BCC cells. 5-FU dosage should be increased to obtain a higher rate of apoptosis in the cultivated BCC cells treated with 5-FU, but this increase might induce cell death in healthy fibroblastic cells.

The results of this study showed that the combination of MMC and 5-FU was effective in inducing BCC cell death; however, it was less effective than MMC. This result suggested that the cell cycle arrest induced by 5-FU inhibited cell cycle progression and reduced the apoptotic effect of MMC on the cultivated BCC cells; the results of the apoptosis and cell cycle analysis in the samples treated with the combination of MMC and 5-FU were consistent with those of the samples treated with 5-FU. These results could be observed because cell cycle arrest occurred early and before cellular apoptosis. The significant decrease in the *CDK6* expression, as one of the components of cell cycle complexes, in the samples treated with 5-FU and in the samples treated with the combination of MMC and 5-FU could verify the inhibitory effect of P21 protein on cell cycle progression complexes^(30-32)^.

Our in vitro results were supported by the favorable clinical outcomes of the presented patient who underwent intralesional injections of the optimal dose of MMC for advanced BCC. However, further clinical studies on the advanced cases of eyelid BCC are needed to prove the reproducibility of our clinical results.

In conclusion, our study demonstrated that the optimal dose of MMC (0.00312 mg/mL) could significantly induce the apoptosis of cultivated human BCC compared with that of the optimal doses of 5-FU (0.312 mg/mL) and their combination. MMC also exerted limited effects on the death of fibroblastic cells. These results suggested that the optimal dose of MMC might be topically or intralesionally used to treat periocular BCC even though it elicited minimal adverse effects on adjacent healthy cells or tissues.
